# Ionic Liquid-Assisted Laser Desorption/Ionization–Mass Spectrometry: Matrices, Microextraction, and Separation

**DOI:** 10.3390/mps1020023

**Published:** 2018-06-19

**Authors:** Hani Nasser Abdelhamid

**Affiliations:** Department of Chemistry, Assuit University, Assuit 71515, Egypt; hany.abdelhameed@science.au.edu.eg

**Keywords:** ionic liquids, matrix-assisted laser desorption/ionization–mass spectrometry, extraction, separation, ionic liquid matrices

## Abstract

Ionic liquids (ILs) have advanced a variety of applications, including matrix-assisted laser desorption/ionization–mass spectrometry (MALDI–MS). ILs can be used as matrices and solvents for analyte extraction and separation prior to analysis using laser desorption/ionization–mass spectrometry (LDI–MS). Most ILs show high stability with negligible sublimation under vacuum, provide high ionization efficiency, can be used for qualitative and quantitative analyses with and without internal standards, show high reproducibility, form homogenous spots during sampling, and offer high solvation efficiency for a wide range of analytes. Ionic liquids can be used as solvents and pseudo-stationary phases for extraction and separation of a wide range of analytes, including proteins, peptides, lipids, carbohydrates, pathogenic bacteria, and small molecules. This review article summarizes the recent advances of ILs applications using MALDI–MS. The applications of ILs as matrices, solvents, and pseudo-stationary phases, are also reviewed.

## 1. Introduction

Room-temperature ionic liquids (RTILs) are salts with melting points at/below room temperature [1,2]. Dialkylimidazolium chloroaluminate may be considered as the first reported RTILs [3]. The typical chemical structure of ionic liquids (ILs) contains a nitrogen or phosphorus organic cation and an organic/inorganic anion [1,2]. These combinations provide a plethora of different RTILs [4]. Suitable combination of anions and cations provide ILs with achiral chiral properties [5]. The properties of the cations and anions moieties govern the chemistry of ILs [6]. The properties of the anion moieties influence the hydrogen bond basicity of ILs, while the properties of the cation moieties influence π–π interactions and in some cases the hydrogen bond acidity. Ionic liquids can be used for green processing industry [7], synthesis of nanomaterials [8], as solvents for clean synthesis [1], catalysis [9], supercapacitors [10], electronic and bioelectronic nose instruments [11].

Ionic liquids have been used in analytical applications [12,13,14,15], including separation [16,17,18], as solvents for headspace gas chromatography [19], liquid chromatography (LC), and capillary electrophoresis (CE) [20], as stationary phases (ILSPs) [21], extraction [22,23,24,25], electrochemical-based sensors [26], and as ion-pairing reagents for the analysis of trace anions using electrospray ionization–mass spectrometry (ESI–MS) in the positive mode [27,28,29]. The addition of imidazolium-based ILs (1-ethyl-, 1-butyl- and 1-hexyl-3-methylimidazolium chloride) to the mobile phase prevents the access of analytes to the free silanols and improves the peak’s shape [30]. Ionic liquids such as 1-butyl-3-methylimidazolium tetrafluoroborate offers green additives compared to other reagents such as triethylamine and shows higher performance for the analysis of β-blockers [31].

Matrix-assisted laser desorption/ionization–mass spectrometry (MALDI–MS) [32,33], and ESI–MS [34,35,36] are soft ionization methods. They can be used for the analysis of thermal labile nonvolatile analytes with very low fragmentation. Matrix-assisted laser desorption/ionization–mass spectrometry was applied for the analysis of pathogenic microorganisms and their lysates [37,38,39,40,41,42,43,44,45,46], endotoxins [47], proteins [48,49,50,51], phosphopeptides [52], biothiols [53], surfactants [54], lipids [55], metals ions [56,57,58], metallodrugs [59], surface chemistry of nanoparticles functionalized with synthetic ligands [60], intermediates for quantum dots formation [61], and small molecules [62]. The ionization of an analyte may be assisted using organic matrices [63], ILs [64,65,66], or nanoparticles [67]. Ionic liquid matrices (ILMs) show high sensitivity in most cases and offer soft ionization with minimal fragmentation of thermal labile biomolecules.

This review summarizes the applications of ILs as matrices and solvents/pseudo-stationary phases for sample microextraction and separation prior to analysis using MALDI–MS. The pros and cons of each application are also discussed.

## 2. Matrix-Assisted Laser Desorption/Ionization–Mass Spectrometry

Laser desorption/ionization mass spectrometry (LDI–MS) is useful for the ionization of analytes that have high absorption at the wavelength of the laser source. The analytes that have no absorption at the laser wavelength require the use of a compound “matrix” that assists the LDI process [68]. The matrix should have a suitable chromophore to absorb the laser irradiation and promote the ionization of the analyte. The matrix and the analyte should be dissolved in a solvent before co-crystallization. The matrix should cause no chemical or thermal fragmentation of the investigated analyte. To circumvent some of these drawbacks, nanoparticles [69,70,71,72,73,74,75,76], and Ils [77] have been used. This review intends to cover the applications of ILs as matrices (Table 1) and solvents for separation and extraction (Table 2) prior to analysis using MALDI–MS. Matrix-assisted laser desorption/ionization–mass spectrometry spectra usually show the singly charged molecular ion of the ionized analyte.

The requirements of conventional organic matrices or ILs, reported below, are similar:

1. Effective ionic liquid matrices (ILMs) usually should have high absorption at the same wavelength of the laser radiation.
2. They should have capability to protonate (positive mode) or deprotonate (negative mode) the target analyte.
3. They should effectively ionize the target analyte.
4. They should effectively ionize all analytes in a mixture without or with minimal ion suppression.
5. They should cause no fragmentation of the analytes.
6. They should form no adduct species with the investigated analytes.
7. They should be miscible with the analyte solution and co-crystalize with the investigated analytes.
8. They should ensure high reproducibility with very low relative standard deviation (RSD) from spot to spot.
9. They should cause no change in the chemical structure of the investigated analyte.
10. They should be cheap and nontoxic.

## 3. Ionic Liquids-Assisted Laser Desorption/Ionization Mass Spectrometry

Armstrong et al. introduced RTILs as matrices for MALDI–MS [78]. Room temperature ionic liquids should have high absorption of the laser irradiation and be able to undergo proton transfer with the analyte. They can be used for a wide range of analytes, including proteins, peptides, oligonucleotides, carbohydrates, lipids, and small molecules [79].

### 3.1. Ionic Liquids-Assisted Laser Desorption/Ionization–Mass Spectrometry Applications for Proteins

Ionic liquids have been applied as matrices for proteins analysis using MALDI–MS. Several ILs based on conventional organic matrices were reported for the analysis of proteins including glycoproteins (Table 1) [80,81]. Ionic liquids were tested for both positive and negative ion extraction modes [82]. Ionic liquids improved the analytical performance and increased the sequence coverage of protein digests obtained using peptide mass mapping (PMM) [83]. They improved protein identification using peptide mass fingerprinting (PMF) [84]. They offered higher matches scores and increased sequence coverage. Ionic liquids consisting of 2,5-dihydroxybenzoic acid (DHB) and aniline were used for *N*-linked glycans derived from human and bovine α1-acid glycoprotein, as well as chicken egg white albumin [85]. Among different ILs, 3,5-dimethoxycinnamic acid triethylamine (SinTri) showed high performance for the analysis of high-molecular-weight proteins such as immunoglobulin G (IgG) [86].

Analysis of proteins using ILMs offers a low limit of detection (LOD), in the range fmol-attomol. Ionic liquids matrices induce no denaturation of the protein or ion suppression, and they can be used to detect proteins in a complex biofluid. The spectra usually show the signal molecular ion peak of the intact protein without or with very low fragmentation. Ionic liquids matrices form no adducts with the analyzed proteins.

### 3.2. Ionic Liquids-Assisted Laser Desorption/Ionization–Mass Spectrometry Applications for Peptides, Carbohydrates, Lipids, and Oligonucleotides

*N*,*N*-diisopropylethylammonium α-cyano-4-hydroxycinnamate, and *N*-isopropyl-*N*-methyl-*t-*butylammonium α-cyano-4-hydroxycinnamate were tested as matrices to assist desorption ionization process for peptides (Table 1) [96]. The storage of peptides in a solution of in CHCA/3-acetylpyridine and CHCA/aniline caused no oxidation compared to a conventional organic matrix CHCA [112]. CHCA/2-amino-4-methyl-5-nitropyridine and CHCA/*N*,*N*-dimethylaniline (CHCA/DANI) offered direct analysis of peptides in tissues [113].

The analysis of phosphopeptides using ILs was investigated. A solid ionic liquid matrix (SILM) consisting of 3-aminoquinoline, CHCA, and ammonium dihydrogen phosphate was used for phosphopeptide analysis [22]. Solid ionic liquid matrix can be used to replace salts and buffer exchange solution that typically follow after phosphopeptide elution from metal oxide affinity chromatography (MOAC) materials [22]. The compounds 1,1,3,3-tetramethylguanidium (TMG) and 2,4,6-trihydroxyacetophenone (THAP), denoted as GTHAP, showed selective ionization of phosphopeptides in the negative ion mode of MALDI–MS [108]. GTHAP showed high tolerance to the presence of salts [108]. Combination of the proton sponge 1,8-bis(dimethyl-amino)naphthalene (DMAN), 6-aza-2-thiothymine (ATT), and diammonium hydrogen citrate (DHC) improved the detection of phosphopeptides, showed lower LOD, reduced signal suppression effects, and improved position-to-position reproducibility [109].

Ionic liquids were applied for the analysis of *N*-linked oligosaccharides [102], disaccharides, sucrose octasulfate, octasulfated pentasaccharides, Arixtra [105], glycans [97], glycosaminoglycans (GAG) polysaccharides [98], and pullulans (polysaccharide polymers consisting of maltotrioseunits, 100 kDa) (Table 1) [103]. Anion-doped liquid matrix G3CA, which consists of p-coumaric acid, and 1,1,3,3-tetramethylguanidine, was applied for the analysis of anion-adducted *N*-glycans [107]. Among different ILMs, *N*,*N*-diisopropylethylammonium α-cyano-4-hydroxycinnamate and *N*,*N*-diisopropylethylammonium ferulate were the best matrices for the analysis of carbohydrates using MALDI–MS [96]. The 1,1,3,3-tetramethylguanidium (TMG) salt of CHCA (G2CHCA) showed no degradation of sulfated oligosaccharides and offered high sensitivity (e.g., 1 fmol) in both ion extraction modes, i.e., positive and negative modes [82]. ILMs consisting of 2-(4-hydroxyphenylazo)benzoic acid (HABA) with 1,1,3,3-tetramethylguanidine or spermine improved the analysis of heparin (HP) and heparin sulfate (HS) that have poor ionization efficiency [110]. DHB-dimethylaniline (DMA) was applied for the analysis of polysaccharides, such as neutral, anionic, methylated, sulfated, and acetylated compounds [114]. ILMs were also reported for mass spectrometry imaging (MSI) of gangliosides [115]. The choice of ILs such as butylamine 2,5-dihydroxybenzoate (DHB-BuN) improved the reproducibility and quantification analysis as well as imaging of oligosaccharides present in soybean and leaves [116].

Ionic liquid matrices showed high ionization efficiency for poor-ionization analytes [110]. ILMs offered no degradation of thermal labile oligosaccharides or DNA oligomers (Table 1) [117]. They showed also high sensitivity in both negative and positive ion extraction modes [82].

Ionic liquid matrices consisting of the ultraviolet (UV)-absorber *p*-nitroaniline with the protonating agent butyric acid were reported for the analysis of lipids (Table 1) [118]. ILMs were applied for the analysis of phospholipids (PLs) [93], lipid imaging [119], and phospholipids imaging in mouse liver and cerebellum tissue sections [120]. ILMs produced high signal intensities, improved spot homogeneity, provided high reproducibility, and showed low LOD (Table 1) [93].

### 3.3. Ionic Liquid-Assisted Laser Desorption/Ionization–Mass Spectrometry Applications for Small Molecules

Conventional organic matrices cause usually interference signals in low mass range (*m*/*z* < 1000 Da) due to self-ionization. The presence of the matrix ion peaks complicates the spectra and may lead to peaks overlap. Triethylammonium α-cyano-4-hydroxycinnamate and diisopropylammonium α-cyano-4-hydroxycinnamate offered direct analysis of 14 pharmaceutical drugs in different formulations, such as coated tablets, noncoated tablets, capsules, and solutions [91]. These peaks can cause also ion suppression of the investigated analytes.

Ionic liquid matrices (ILMs) of mefenamic acid and bases (aniline (ANI), pyridine (Pyr), dimethylaniline (DMANI), and 2-methylpicoline (2-P)) were applied for the analysis of small molecules, including drugs, carbohydrates, and amino acids [106]. ILMs were also applied for the analysis of aflatoxins B1, B2, G1, and G2 [99], and carbonaceous compounds [121]. The compound 2-aminopentane (AP)-CHCA was used for quantitative analysis of *N*-acyl homoserine lactones (AHL) in the low pico-molar range, with lower limits of quantification (LOQ) from 1–5 pmol for different AHL [92].

Selected ammonium-, phosphonium-, and sulfonium-based ILMs and bis(trifluoromethylsulfonyl)imide as a counter ion were applied for the analysis of lubrication specimens [122]. Formation of pyrylium salts in brain tissue sections offered the imaging of primary amines [123]. The 2,4-diphenyl-pyranylium ion can be used for derivatization of primary amines such as dopamine (DA) in coronal tissue sections.

The analysis of small molecules using ILMs is promising. ILMs show very few or no interference peaks in the low mass range (<1000 Da). The choice of a suitable base conjugate may suppress the ion peaks of the conventional organic matrices, with low or no negative impact on the ionization of the target analytes. Thus, ILMs show interference-free spectra for the analysis of small molecules. ILMs show no adducts peaks, and that leads to an accurate determination of the molecular weight. The ionization efficiency of small molecules usually is higher than the ionizability of molecules with large molecular weight. ILMs show effective ionization of both species in a mixture without any ion suppression for large-molecular-weight molecules.

### 3.4. Ionic Liquid-Assisted Laser Desorption/Ionization–Mass Spectrometry Applications for Polymer and Pathogenic Bacteria

*N*,*N*-diisopropylethylammonium *α*-cyano-4-hydroxycinnamate (DEA-CHCA) was used for the characterization of polar biodegradable polymers [124]. DEA-CHCA offered the greatest signal with the smallest laser power and negligible polymer degradation. Ionic liquid matrices were also applied for aliphatic biodegradable photoluminescent polymers [125]. They were also used for the analysis of lubricant residue polydimethylsiloxane standards (PDMS2000, PDMS6000, PDMS9000, PDMS17000) of condom lubricants in biological fluids [94]. A comparison between six conventional matrices with and without potassium and six ILMs, namely, trans-2- [3-(4-tert-butylphenyl)-2-methyl-2-propenylidene]malononitrile (DCTB) DCTB–triethylamine (TEA), DCTB-butylamine (BA), DHB-BA, CHCA-TEA, CHCA- *N*,*N*-diisopropylethylamine (DEA), and sinapinic acid (SA)-TEA, was reported for the analysis of poly(ethylene glycol) (PEG), polytetrahydrofuran (PTHF), and poly(methyl methacrylate) (PMMA) [126]. The authors reported that common organic matrices are superior to ILMs for some polymer species [126]. They claimed that the low efficiency of ILMs is due to the lower UV absorption values [126]. Ionic liquid matrices were also used for imaging synthetic polymer samples [127].

The analysis of polymers using MALDI–MS is challenging because of the poor ionization and high molecular weight of polymers. Ionic liquid matrices should cause no degradation of the polymers and produce a single peak corresponding to the molecular ion. Thus, the parent molecular ion peak of the intact polymer can be detected. They should produce a sharp peak with very small width at half maximum.

Abdelhamid et al. reported two series of ILMs using organic matrices sinapinic acid and 2,5-DHB in conjugation with the organic bases aniline (ANI), dimethylaniline (DMANI), diethylamine (DEA), dicyclohexylamine (DCHA), pyridine (Pyr), and 2-picoline (2-P), 3-picoline (3-P) for the analysis of pathogenic bacteria species [128]. ILMs enhanced the signals for bacteria biomolecules species and improved spot homogeneity. A series of ILMs of mefenamic acid and bases (ANI, Pyr, DMANI, and 2-P) was used to detect bacteria toxins without separation or any pretreatment steps [106]. 1-butyl-3-methylimidazolium hexafluorophosphate was used to capture bacteria cells from yogurt samples prior to analysis using MALDI–MS [129]. The separation procedure is simple, and offers higher sensitivity compared to the direct analysis [129].

The analysis of intact bacteria using ILMs is promising for biomedical studies. The analysis of bacteria cells using MALDI–MS requires a very short time (<5 min) compared to conventional methods such as bacteria culture that needs several days. The method offers high-throughput analysis and can be used in real analyses in hospitals and medical clinics. The use of statistical analyses combining MALDI–MS showed high accuracy and good precision for bacteria identification. The analysis of bacteria using MALDI–MS is simple, fast, accurate, and high-throughput. However, the quantification analysis is a challenge. The bacteria mass profiles are usually influenced by time and sample preparation. A few ILMs appeared suitable for the analysis of proteins or biomarkers with high molecular weights.

### 3.5. Imaging Using Ionic Liquid Matrices

Ionic liquid matrices were applied for MALDI–MS imaging (MALDI–MSI) of lipids [119], phospholipids in mouse liver and cerebellum tissue sections [120], protein distribution and identification within formalin-fixed paraffin-embedded (FFPE) tissue sections [130], gangliosides [115], and oligosaccharides in soybean and leaves [104]. MALDI–MSI using ILMs allows the study of protein distribution and identification directly within tissue sections [130], and improve the reproducibility of the results [104].

### 3.6. Quantitative Analysis Using Ionic Liquid Matrices-Assisted Laser Desorption/Ionization-Mass Spectrometry

Quantification analysis using MALDI-MS is a challenge due to the signal fluctuations. A few studies were reported for quantification analysis using MALDI–MS (Table 1). For successful quantification analysis, Wang and Giese summarized 18 recommendations for the quantitative analysis of small molecules using MALDI–MS [131]. ILMs offered many advantages that are absent in conventional organic matrices. Thus, they are promising for quantification analysis.

Tholey et al. reported the quantification analysis of a low-molecular-weight compound (i.e., glutamine) using ILMs and an internal standard [111]. ILMs were tested as matrices for the quantification of oligodeoxynucleotides (ODNs), peptides, small proteins [87], sialylated glycans [100], *N*-acyl homoserine lactones (AHL) [92], and phosphatidylcholine (PC) in mouse brain tissue [89]. Out of 27 combinations of acids and bases, ILMs of CHCA and 3-aminoquinoline or *N*,*N*-diethylaniline were the best choices for peptide quantification [95].

The quantification analysis of peptides without internal standards was also reported [132]. The data showed a linear correlation between peptide amounts and signal intensities. This advantage offered the monitoring of the time-dependent evolution of substrates and products in trypsin-catalyzed digests of single peptides and peptide mixtures [132]. The quantification analysis of a mixture shows that ILMs effectively ionize the mixture molecules without any observable ion suppression.

## 4. Factors Influencing the Analysis Using Ionic Liquid Matrices

There are several key parameters that influence the analysis using ILMs for MALDI–MS. Thus, there is no general rule to predict the performance of the ILMs. Furthermore, the gaps in knowledge about MALDI ionization behavior make the prediction of ILMs performance for a specific sample difficult a priori. The following points highlight some of these influencing parameters.

### 4.1. Types of Ionic Liquid Matrices and Analytes

The performance of ILMs varies depending on the types of ILMs and analytes. Thus, some reports showed comparable or increased sensitivities [90,113,127], whereas others studies reported a decrease in the sensitivities [129,133]. The best ILMs can be used for the selective analysis of species such as phosphopeptides in peptide mixtures and in proteolytic digests using negative ion mode [108]. Cool and high salt-tolerant ILMs G3THAP was considered as the best choice for the preferential ionization of phosphopeptides for negative ion mode [108]. G3THAP showed very low ion suppression caused by nonphosphopeptides for phosphopeptides [108].

### 4.2. Preparation of Ionic Liquid Matrices

Ionic liquid matrices are usually prepared by mixing the solution of a conventional organic matrix with molar equivalents of a counter base. The type and acid:base molar ratio influence the performance of ILMs. Thus, these parameters require optimization.

The physicochemical properties of the base conjugates influence the performance of ILMs. Thus, the selection of the optimal base conjugate is critical. The effect of the base type using pyridine, aniline, *N*,*N*-dimethylaniline, and *n*-butylamine (BuN) for the conventional organic matrix was studied for the analysis of oligosaccharides [104]. The authors found that BuN improved the performance of DHB compared to the other bases [104].

The molar ratio of organic matrix to base influences the performance of ILMs. The analysis of *N*-acyl homoserine lactones (AHL) using ILMs of CHCA and mono/di-amount of 2-aminopentane (AP) werereported [92]. The data showed that the ILMdi-AP-CHCA containing a double molar excess of base offered the best results in terms of quality of the MALDI spectra [92]. However, it was found that the ILM consisting of a TMG–THAP (G3THAP) ina molar ratio of 3:1 was the best choice for high performance for phosphopeptide analysis [108].The acid to base molar ratio affects the ionization of the investigated analyte as protonated or as adduct ions peaks [92]. The acid to base molar ratio from stoichiometric to nonstoichiometric can be also investigated to improve the fluidic characteristics of ILMs [95].

### 4.3. Sample Preparation

There are several methods for sample preparation for MALDI–MS [116]. Most of ILMs do not solidify after solvent evaporation [23]. Thus, they tend to form homogeneous spots and offer high reproducibility compared to conventional organic matrices. However, the choice of sample preparation influences the performance of the ILMs [128]. Abdelhamid et al. tested several methods, including the dried-droplet method and the double-layer method [128]. They observed that both methods were not suitable for the analysis of bacteria species. They found that the addition of a drop of ILMs on the two spots of bacteria species improved the performance of the tested ILMs for the analysis of pathogenic bacteria [128]. The sample preparation influences the spot properties and the analysis performance.

### 4.4. Solvent

The choice of solvent influences the deposition and crystallization for dried-droplet sample deposition [134]. The solvent affects the evaporation rate of the matrices and the spot homogeneity. Water is the common solvent; however, solvents such as methanol, ethanol, or acetonitrile were also investigated.

### 4.5. Additives

The additives, such as potassium trifluoroacetate (KTFA), which served as a cationization agent [126], play also a key role in the performance of ILMs. The excess of KTFA dissipated any overrepresentation of high-molecular-weight polymer species [126]. Other additives, including trifluoroacetic acid (TFA), phosphoric acid (PA) [135], and ammonium dihydrogen phosphate (ADP) [136], were also reported. These additives improved the ionization efficiency of ILMs. Furthermore, it was reported that the addition of PA to G3THAP reduced the background noise for the analysis of phosphopeptides [108]. The role of these additives is unknown, and in most cases their choice is a trial-and-error experiment.

### 4.6. Impurities

The presence of impurities in a sample, especially in biological samples such as tissues, influences the ionization performance of ILMs [89]. Tissues are often contaminated with undesired reagents, such as alkali metal salts, choline and its derivatives, etc., that may cause ion suppression. Thus, impurities render the quantification of phosphatidylcholine (PC) in mouse brain tissue a difficult task [89]. Further steps to eliminate most of the polar contaminants and some nonpolarones are usually necessary. Park et al. overcame this challenge by washing the tissue samples with water [89]. The impurities consisting of alkali species are inevitable and may affect the ionization of the target analyte and thus produce ion peaks as alkali adducts.

### 4.7. Instrumental Parameters

Instrumental parameters, including the type of laser, detector, mode of detection (positive or negative modes), and detectors, affect the performance of ILMs (Table 1).

## 5. Principles and Mechanisms of Ionization Using Ionic Liquid Matrices

The mechanism of the ionization using conventional organic matrices or ILMs is unknown. However, there are several proposed mechanisms to explain the ionization process [137,138]. It is important to keep in mind that the ionization using MALDI–MS cannot be explained using a single mechanism. However, the ion formation mechanism can be classified into:

(1) Primary ion formation, including multiphoton ionization (MPI), pooling, excited-state proton transfer (ESPT), disproportionation, thermal proton transfer [139,140], and spallation.
(2) Secondary ion formation, including H^+^ transfer, e^-^ capture and H^+^ transfer, cationization, e^-^ transfer, and ejection [138].
(3) The ’’Lucky Survivor” model; this model claims that the ionization take places in the solution, and the ionized species retain their solution-state charge and exist as preformed ions within the solid state matrix [141].
(4) Ionization due to solid-to-gas phase transition: this mechanism was proposed for the ionization induced in infrared (IR)-MALDI [142]. This model was also suggested for UV-MALDI that involves ionization without an obvious contribution from electronic excitation [142].

The ion formation mechanism depends on many parameters including the laser properties [143], such as wavelength (infrared or UV-laser), photon energy, energy density (J/cm^2^), laser irradiance (W/cm^2^), incident angle of the laser beam, laser exposure time, matrix type, analyte ionizability, additives, impurities, and sample preparation methods.

**Figure 1:**
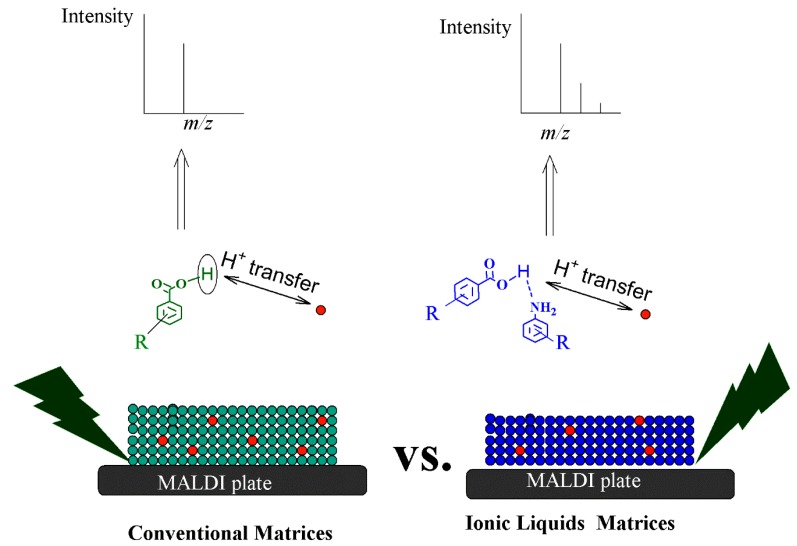
Proton transfer between conventional organic matrices (left) or ionic liquids matrices (right) and an analyte for matrix assisted laser desorption/ionization mass spectrometry.

These proposed mechanisms were reported for conventional organic matrices that should have absorption matching the wavelength of the laser. They are also valid for ILMs. However, it is important to mention that the proton transfer can be assisted by the base moieties, as shown in Figure 1.

## 6. Advantages of Ionic Liquid as Matrices

Ionic liquid matrices offer several advantages compared to conventional organic matrices and nanoparticles. ILMs have shown higher sensitivity compared to the corresponding organic matrices. In a study, the presence of a weak base enhanced the sensitivity [144]. ILMs offer high signal-to-noise ratios, reduction of chemical noise, and reduced formation of alkali adducts [145]. The detection sensitivity using ILMs is in the range of fmol-attomol (Table 1). ILMs, in most cases, require no sample pretreatment or preconcentration.

Ionic liquid matrices improve the ionizability of analytes with poor ionization efficiencies. Polyanionic oligosaccharides, including dermatan sulfate (DS) and chondroitin sulfate (CS), have poor ionization efficiencies. Thus, they usually require derivatization to improve their ionization. A guanidinium salt of CHCA offered the direct analysis of underivatized DS and CS oligosaccharides up to a decasaccharide. ILMs are suitable for the analysis of a mixture containing oligosaccharides with different numbers of sulfogroups [146].

Ionic liquid matrices can be also used for species that face ionization suppression in the presence of high ionization analytes. The compound 1,1,3,3-tetramethylguanidinium 2,4,6-trihydroxyacetophenone (GTHAP) was used for the analysis of glycopeptides and glycans, in the presence of peptides [147]. ILMs overcame the well-known ionization suppression of the carbohydrates.

Adducts formation of analytes with alkali ions sodium or potassium sometimes causes ambiguity. It has been reported that the analysis using ILMs showed no peaks related to adduct species. Water-immiscible ILs was the best choice for analytes with low molecular weight. In contrast, proteins showed the best results in water-miscible ILMs [148].The ionization efficiency of ILMs could be improved by the addition of matrix additives, including trifluoroacetic acid (TFA) and phosphoric acid [135].

Because of their high solvation capabilities, ILMs dissolve a wide number of different analytes. ILMs offered homogenous spots compared to conventional organic matrices [149]. Thus, they offered better shot-to-shot reproducibility. The sample homogeneity can be increased by additives such as TFA or phosphoric acid. The formation of homogenous spots decreases the time employed to search hot spots. Thus, ILMs can be used for microfluidic sample deposition [23]. ILMs showed no solidification during the spotting and caused no formation of sweet or hot spots.

The fragmentation of large molecules may be caused by the high laser energy or the acidity-basicity of the conventional matrices. ILMs showed low or no fragmentation [78]. Conventional organic matrices cause thermal fragmentation of polyanionic oligosaccharides through the loss of sulfur trioxide (SO_3_) groups [90]. Thus, oligosaccharides are usually derivatized to suppress the fragmentation. A guanidinium salt of CHCA allowed the direct analysis of underivatized oligosaccharides with very low fragmentation. Suitable ILMs could significantly suppress fragmentation [90].

**Figure 2:**
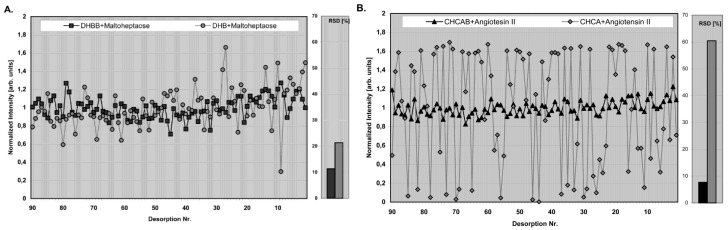
(**A**) Plot of normalized [M + Na]^+^ ion intensities of the oligosaccharide maltoheptaose with 2,5-DHB butylamine (DHBB) (black squares) and DHB matrix (grey circles). (**B**) Resulting [M + H]^+^ ion intensities from a human angiotensin II preparation with α-cyno-4-hydroxycinnamic acid-butylamine (CHCAB) (black triangles) and CHCA matrices (grey squares). Figure reprinted with permission from Reference [86].

Conventional organic matrices lack high reproducibility compared to ILMs (Figure 2). The ion intensities of the oligosaccharide maltoheptaose using 2,5-DHB butylamine (DHBB, black squares) showed stable signal intensities and low relative standard deviation compared to the corresponding signals obtained with a conventional matrix DHB (grey circles) (Figure 2) [86].

The quantification analysis using MALDI–MS requires: (1) controlled and stable total ion current (TIC); (2) constructing a calibration curve by plotting the ion ratio versus the analyte concentration; (3) keeping the matrix suppression below a critical value [95]. Compared to conventional organic matrices, ILMs showed a stable ion current and allowed the quantification analysis for several analytes, including peptides [88], pyranose oxidase [150], N-acylhomoserine lactones (AHL) [92], and the environmental neurotoxin β-*N*-methylamino-L-alanine (L-BMAA) in brain tissue sections [123]. DHB-N-methylaniline (N-MA) and DHB-N-ethylaniline (N-EA) were used for the qualitative and quantitative analysis of carbohydrates [101].

Ionic liquid matrices were applied for several analytes, including proteins, peptides, oligonucleotides, and phospholipids. They offer the broadest applicability to a wide range of biomolecules [86]. They can ionize the different analytes in a mixture without observable ion suppression.

The melting point of ILs is below room temperature. Thus, ILs remain in liquid state even after the solvent evaporates [151]. The liquid state of ILMs allowed the in situ extraction of analyte species and improved the performance of ILMs [121].

Ionic liquid matrices can be used as matrices, modifiers, additives, and co-solvents [81,152]. These applications ensure high performance of ILMs. They reduce the use of chemicals and provide green technologies. They can be used for applications, such as microextraction and separation, which improve the analysis for MALDI–MS.

Ionic liquid matrices (ILMs) have a low vapor pressure and show high stability under vacuum. They show a very low tendency to sublimation. They offer homogeneous spots under vacuum, high ion peak intensity, and clean spectra [78].

## 7. Applications of Ionic Liquids for Microextraction Using Matrix Assisted Laser Desorption/Ionization Mass Spectrometry

Ionic liquids can be also applied as solvents for microextraction using MALDI–MS (Table 2). The use of ILs improves the detection using MALDI–MS. The microextraction improves sample spotting, enhances the sensitivity, offers higher selectivity, and decreases ion suppression caused due to undesirable species in a mixture.

α-cyno-4-hydroxycinnamic acid-butylamine (CHCAB) was used as a solvent and a matrix for extraction and ionization of phospholipids from food samples (soybean) using dispersive liquid–liquid microextraction (DLLME) prior to analysis using MALDI–MS (Table 2) [153]. The data showed 8–125-fold improvements in signal intensities after microextraction (Table 2).

Ionic liquids have affinity for coordination to metal cations. Uranyl nitrate was extracted using tributylphosphate (TBP) as UO_2_(NO_3_)_2_·2TBP prior to analysis using ESI–MS and MALDI–MS (Table 2) [154]. The analysis showed high sensitivity and simple sample preparation and can be extended to additional metal ions.

**Figure 3:**
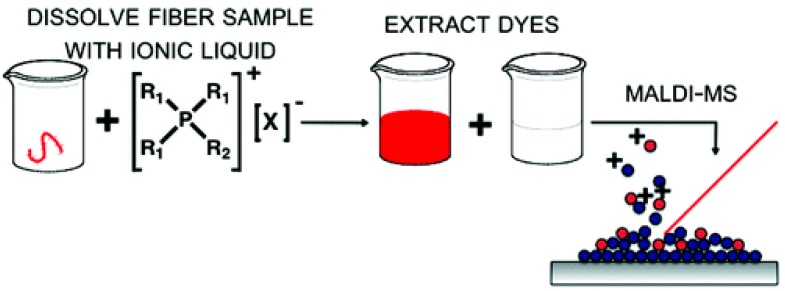
Extraction procedure of dyes using tetraalkylphosphonium (PR_4_^+^)-based ionic liquids. Figure reprinted with permission from reference [156].

Room temperature ionic liquids (RTILs) tetraalkylphosphonium (PR_4_^+^) cations ferulate (FA), CHCA, and 2,5-DHB anions allowed the separation of dyes from textiles, the extraction of dyes from aqueous solutions, and the identification of dyes using MALDI–MS in a single experimental step (Figure 3) [156]. The use of PR_4_^+^-based ionic liquids allowed the detection of small-molecule dyes without the addition of a traditional solid matrix.

Ionic liquids offered nondestructive separation of dyes from wool [157]. The method requires a small volume, offers high-throughput analysis for accelerated threat-response times, and requires no matrices for laser desorption/ionization process.

**Figure 4:**
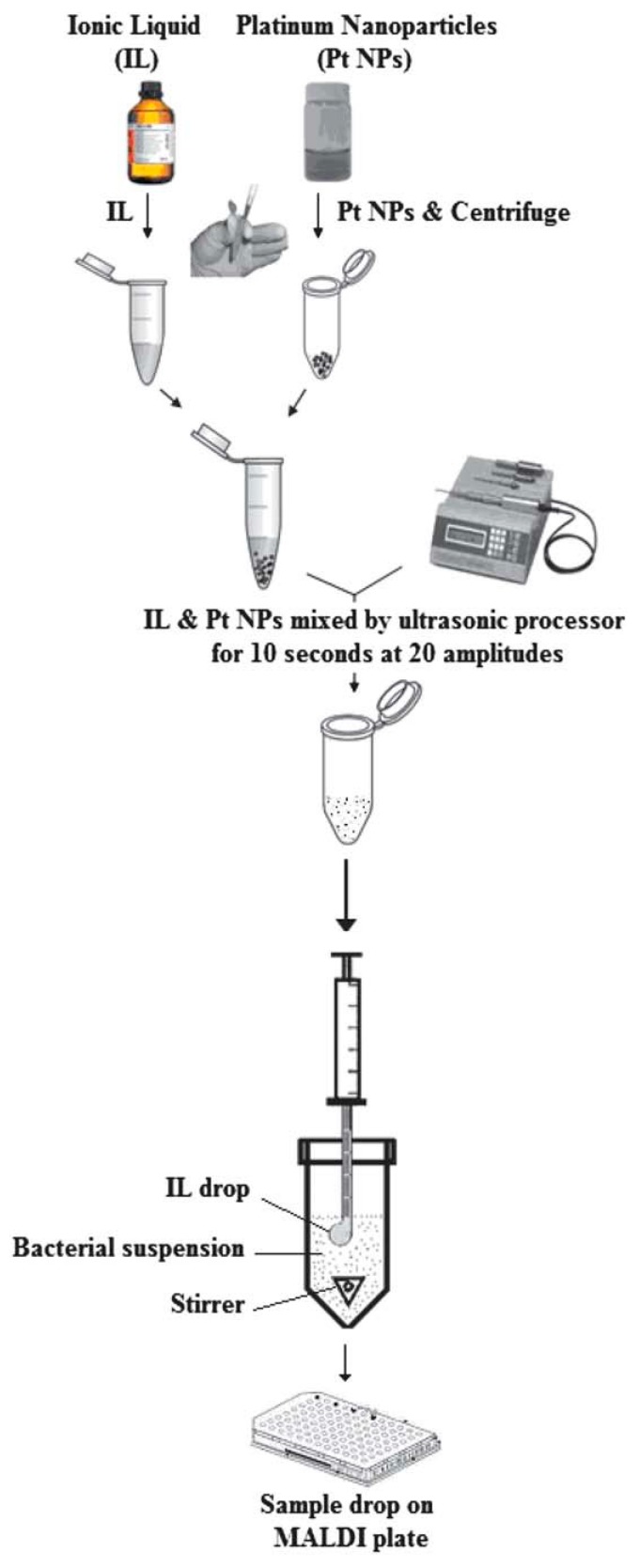
Single-drop microextraction (SDME) using ILs for the extraction of pathogenic bacteria from aqueous suspensions. Figure reprinted with permission from reference [158].

The single-drop microextraction (SDME) approach using ILs allowed the extraction of pathogenic bacteria from aqueous samples for characterization by MALDI–MS (Figure 4). Platinum nanoparticles mixed in 1-butyl-3-methylimidazolium hexafluorophosphate were used in an extraction drop (Table 2) [158]. The method requires simple mixing of ILs and platinum nanoparticles (Pt NPs) (Figure 4). The separated drop is sufficient for analysis using MALDI–MS. 1-butyl-3-methylimidazolium hexafluorophosphate was applied to capture bacteria from yogurt samples prior to analysis using MALDI–MS [129].

The microextraction of pathogenic bacteria using ILs offered high sensitivity with low LOD [158]. Microextraction using ILs improved the sample preconcentration and showed no negative impact on bacteria identification. It improved the statistical analysis and showed high accuracy compared to the direct analysis. This process shows no cytotoxicity and no influence on the bacteria counts. Microextraction prior to the analysis using MALDI–MS increased the number of the protein peaks of intact cells without the need of complicated equipment.

## 8. Advantages of Ionic Liquids for Microextraction

The combination of organic matrices and organic bases offers custom-synthesized ILs (Table 2). They can be custom-synthesized to be either miscible or immiscible with water or organic solvents. Thus, they are useful for liquid–liquid microextraction (LLME). The structure and functionality of the cations and anions control the water solubility of the RTIL. Most water-immiscible RTILs contain either PF_6_^–^ or bis[(trifluoromethyl)sulfonyl]imide anions.

Microextraction using ILs offers a nondestructive extraction of target analytes from a complicated mixture. The procedure of microextraction usually requires a small volume of ILs and other solvents, provides high-throughput analysis, and requires no addition of MALDI matrices or especial equipment.

## 9. Applications of Ionic Liquids for Analyte Separation Using Matrix Assisted Laser Desorption/Ionization Mass Spectrometry

Separation of a target analyte is usually needed before the analysis of a complicated mixture (Table 2). The separation procedure prevents ion suppression caused by highly ionizing species. It is also used to resolve the species that have the same molecular weight.

The viscous ionic liquid 3-aminoquinoline/CHCA (3-AQ/CHCA) was used to separate the buffer components, peptides, and oligosaccharides of a solution (Table 2) [159]. The method is simple, cheap, needs no external matrices, and offers green technology (Table 2).

**Figure 5:**
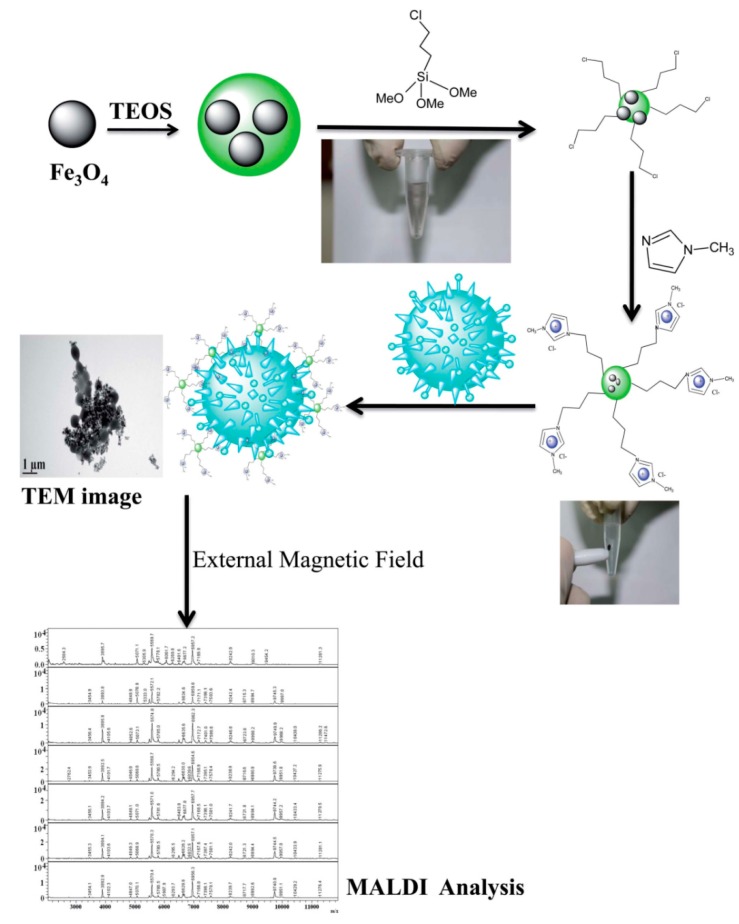
Schematic illustrations of the preparation of cationic IL-modified magnetic nanoparticles (CILMS) and the capture of bacteria by the magnetic nanoparticles. Reproduced from reference [160] with permission from The Royal Society of Chemistry. TEOS: Tetraethoxysilane; TEM: Transmission electron microscope.

Cationic IL-modified magnetic nanoparticles (CILMS) were used to separate pathogenic bacteria prior to identification using MALDI–MS (Figure 5) [160]. The separation procedure employed an external magnetic field and required a very short time for extraction. The presence of positive charges on the surface of the magnetic nanoparticles (MNPs) strengthened the interactions with the negative charges of the bacteria cell walls. CILMS nanoparticles enhanced the signal-to-noise ratios and offered low LOD.

Thin-layer chromatography (TLC) was used for the fast separation of small compounds prior to direct on-spot analysis using MALDI–MS [161]. The method using UV-absorbing ILMs offered nearly “matrix-free” mass spectra.

### Advantages of Ionic Liquids for Separation

Ionic liquids have unique properties, such as negligible vapor pressure, good thermal stability, tunable viscosity, and miscibility with water and organic solvents, as well as good extractability of various organic compounds and metal ions [162]. Analyte separation using ILs is fast, requires no special equipment, and is compatible with MALDI–MS. ILs can be used as solvents and matrices at the same time.

## 10. Challenges and Remarks

The analysis of nonvolatile and thermal labile biomolecules using MALDI–MS is promising for clinical and real-sample analyses. However, there are no general rules for predicting the suitability of ILs as a matrix. The optimization of ILs is a trial-and-error experiment. Therefore, it is often necessary to test a range of ILMs with different sample preparation methods to find the suitable conditions. Furthermore, the base:acid ratio may affect the material performance. The optimization of the molar ratio is highly required.

The applications of ILs for microextraction and separation are still in the infancy stage. Further investigations are highly required. The optimization of microextraction and separation is highly required for high efficiency. The presence of impurities in ILs may influence their properties. These impurities may cause ion suppression or interference peaks in the spectra. New synthesis methods with high purity are highly demanded.

The role of additives such as TFA or phosphoric acid is not fully understood. These additives can improve the performance of ILs and can add more functions to the ILs. The presence of these additives can improve sample preparation, increase sensitivity, and may offer better selectivity. They can also reduce the fragmentation of thermal labile species and reduce the laser energy.

## Figures and Tables

**Table 1:** Chemical compositions and applications of ionic liquids (ILs) for matrix-assisted laser desorption/ionization–mass spectrometry (MALDI–MS) as matrices.

Acid	Base	Analyte	Conditions	Low Limit of Detection (LOD, pmol)	Linear Range (pmol)	Ref.
CHCA	1-methylimidazole, aniline, pyridine, *N*,*N*-diethylamine, triethylamine, tripropylamine, tributylamine	ODNs, proteins 5′-d(CTTTCCTC) and 5′-d(TCTTCCCTT), bradykinin, Tyr-bradykinin, substance P, melittin, and bovine insulin	Voyager DE-RP mass spectrometer Nitrogen laser (337 nm, 3 ns pulse) Linear positive-ion mode The acceleration voltage was 20 kV Grid voltage was 95%, Guide-wire voltage was 0.1% Delay time was 200 ns		2 μM to 50 μM	[87]
	3-aminoquinoline	Tetrapeptide RFDS, bradykinin fragment 1-7, angiotensin I, substance P, Glu-fibrinopeptide, ANP 104-123, ACTH 18-39, Somatostatin, and ACTH 7-38	Waters Micromass Q-TOF Premier Spot size of about 200–300 μm	1	0.001–2	[88]
		Phosphatidylcholine (PC) in mouse brain tissue	Lasertechnik Berlin Nitrogen laser (337 nm, a pulse energy of 1.5 µJ)	30	1–100	[89]
	*n*-butylamine, *N*,*N*-diethylaniline, aniline, *N*,*N*-diethylaniline	Bradykinin, substance-P, melittin, allatostatin IV oligonucleotide 5′*-GGATTC-*3′ phosphatidylcholine, L-α-phosphatidylcholine-β-palmitoyl-oleoyl, ([PC 16:0, 18:1]), and phosphatidylethanolamine, 1-2,dioleoyl-sn-glycerol-3-phospho-ethanolamine, ([PE 18:1, 18:1])	MALDI FTMS spectra were collected with a 3 tesla FTMS Nitrogen laser (337 nm) Full power (60–70 μJ). The laser spot size is 0.196 mm^2^	5000		[90]
	Triethylamine, diisopropylammine	Drugs	Micromass MALDI-LR^®^ Nitrogen laser (337 nm) A pulse voltage of 2.5 kV; a delay extraction of 500 ns; an accelerating voltage of 15 kV Reflectron voltage of 2 kV			[91]
	2-aminopentane (AP)	N-acyl homoserine lactones (AHL)	AB SCIEX MALDI TOF/TOF 5800 mass spectrometer Nd:YAG laser (355 nm) A pulse rate of 400 Hz Accumulating 2000 shots	0.125–5		[92]
	1-methylimidazole, aniline, pyridine, tripropylamine, tributylamine	Phosphatidylcholine (PC), phosphatidic acid (PA), phophatidylethanolamine (PE), serine (PS), glycerol (PG), and inositol (PI)	A Voyager DE-STR mass spectrometer Nitrogen laser (337 nm) Acceleration of 20 kV, Delay time of 400 ns 200 laser shots	127 × 10^3^		[93]
	*N*,*N*-diisopropylethylammonium	Polymers and additives found in lubricant residues	Bruker Daltonics AutoFlex Nitrogen laser (337 nm)	0.5% and 0.003% lubricant in biological fluid		[94]
	3-aminoquinoline, *N*,*N*-diethylaniline	Peptides Y_5_R, Y_6_, and substance P arginine, imipramine, and serotonin	MNL100 Lasertechnik Nitrogen laser (337 nm)	10^−2^	10^−2^–10^3^	[95]
	*N*,*N*-iisopropylethylammonium, *N*-isopropyl-*N*-methyl-*t-*butylammonium, *N*-isopropyl-*N*-methyl-*N*-ter*t*-butylammonium, *N*,*N*-diisopropylethylammonium	Bradykinin, polyethylene glycol 4600, insulin, cytochrome *c*, bovine serum albumin (BSA), catalase, urease, dextran enzymatic synthesis, *Saccharomyces cerevisiae*.	Bruker Autoflex and Bruker Flex Analysis Software Nitrogen laser (337 nm)	50–100		[96]
	3-aminoquinoline (3-AQ)	Glycan	AXIMA-Resonance UV-MALDI Nitrogen laser (337 nm) 3 ns pulse width The maximum laser pulse rate is 10 Hz	1 × 10^−3^		[97]
	1-methylimidazolium	Glycosaminoglycan (GAG) polysaccharides	4800 MALDI TOF/TOF™ Analyzer Nitrogen laser (337 nm) Reflectron negative mode Accelerating voltage 1 kV			[98]
CHCA	Triethylamine	Aflatoxins B_1_, B_2_, G_1_, and G_2_	Micromass Nitrogen laser (337 nm) Reflectron and positive ion modes Pulse voltage, 2450 V Delay extraction 100 ns Acelerating voltage, 15 kV Reflectron voltage, 2 kV	0.05		[99]
2,5-dihydroxybenzoic acid (DHB), CHCA, Sinapic acid	Butylamine, Triethylamine	Glycoconjugates, peptides, and proteins oligosaccharides, polymers desialylation of sialylactose, sialidase from *Clostridium perfringens*	A Voyager DE-STR MALDI-TOF MS Nitrogen laser (337 nm) Acceleration voltage, 20–25 kV Grid voltage, 95% and 72% Guidewire voltage, 0.05% Extraction delay time, 300–550 ns	0.3–2.5		[86]
CHCA and ferulic acid	*N*,*N*-iisopropylethylammonium, *N*,*N*-diisopropylethylammonium, *N*-isopropyl-*N*-methyl-*N*-ter*t*-butylammonium, *di*(2-aminopentane)	Mannan, β-Cyclodextran dextran, polyethylene glycol 4600	Bruker Autoflex mass spectrometer	10^3^		[96]
DHB	Aniline, *N*,*N*-dimethylaniline (DMA)	Sialylated Glycans	Bruker Biflex IV MALDI-TOF Nitrogen laser (337 nm) Positive-ion extraction mode Accelerating voltage 9.3–20 kV	30		[100]
	*N*-methylaniline (*N*-MA), *N*-ethylaniline (*N*-EA)	Maltohexaose, maltoheptaose, dextran 2000 (D2000) and dextran 4000 (D4000), 1-Kestose (GF2), nystose (GF3) and 1,1,1-kestopentaose (GF4)	Bruker UltrafleXtreme™ mass spectrometer Nd:YAG laser (355 nm) Positive and reflectron mode Accelerating potential 20 kV	0.01	10–80	[101]
	*N*,*N*-dimethylaniline (DMA)	*N*-linked oligosaccharides Ovalbumin (chicken egg white albumin), maltohexaose, maltoheptaose, dextran standard 1000	Bruker Biflex IV Nitrogen laser (337 nm) Positive ion reflecting mode	7–22.4	0.7–22.4	[102]
	Butylamine	Pullulans Pul-5900 5.9 Pul-11,800 11.8 Pul-22,800 22.8 Pul-47,300 47.3 Pul-112,000 112.0	AXIMA-LNR Nitrogen laser (337 nm) Accelerating voltage of 20 kV 200 laser shots	0.8–4.4		[103]
		Oligosaccharides sucrose (disaccharide), raffinose (trisaccharide), stachyose (tetrasaccharide), ß-cyclodextrin, L-proline, D,L-pyroglutamic acid, L-arginine hydrochloride, D,L-tyrosine, angiotensin II, reduced glutathione and sunflower oil	SolariX 7.0 Fourier transform ion cyclotron resonance mass spectrometry (FT-ICR-MS) Nd:YAG laser (355 nm)	38	340–555	[104]
CHCA *p*-coumaric	1,1,3,3-tetramethylguanidium (TMG)	Sulfated/sialylated/neutral oligosaccharides	AXIMA-QIT Nitrogen laser (337 nm) 100 laser shots for each analysis	0.001		[82]
CHCA and DHB	1-methylimidazolium	Sucrose octasulfate, and an octasulfatedpentasaccharide, Arixtra	TofSpec2E MALDI-TOF Nitrogen laser (337 nm) Reflectronand positive mode Accelerating voltage 20–26 kV	8–40		[105]
Mefenamic acid	Aniline (ANI), Pyridine (Pyr), Dimethyl aniline (DMANI), 2-methyl picoline (2-P))	Drugs, carbohydrate, and amino acids.	Bruker Microflex IV Nitrogen laser (337 nm)	1–20		[106]
*p*-coumaric acid	1,1,3,3-tetramethylguanidium (TMG)	Anion adducted *N*-glycans	µFocus MALDI plate TM 700 µm Nitrogen laser (337 nm) 100 laser shots	0.001 for NO_3_^–^, 0.001 for BF_4_^–^		[107]
THAP		Phosphopeptides	Autoflex speed TOF/TOF mass spectrometer Nd:YAG laser (355 nm) Laser energy 5–10% above the ionization threshold 500 laser shots The delayed extraction time 150 ns			[108]
ATT	DMAN		Waters MicroMX MALDI Nitrogen laser (337 nm) Tubevoltage12 kV Reflectronvoltage5.2 kV Anode voltage 5 kV accelerate voltage 20 kV, MCP detector Voltage19.5 kV Extraction delay 500 ns	5 × 10^−4^	0–100	[109]
HABA	1,1,3,3-tetramethylguanidine Spermine	Polysulfated carbohydrates such as heparin (HP) and heparan sulfate (HS)	Biosystems Voyager-DE Pro STR MALDI-TOF Nitrogen laser (337 nm) Pulsed at a 20 Hz frequency Negative ion reflector mode Accelerating potential of −20 kV	67		[110]
DHB CHCA SA	Tributylamine (TBA), Pyridine (Py), 1-methylimidazole(MI)	Arabinose, biotin, thiamine, NAD, ascorbic acid, a-ketoglutarate, ATP	Bruker Reflex III Nitrogen laser (337 nm) Energy of 400 mJ/pulse Accelerated voltage 20 kV	0.01	0.25–2.5	[111]

Notes: ATP: Adenosine 5-triphosphate; ATT: 6-aza-2-thiothymine; CHCA: α-Cyano-4-hydroxycinnamic acid; DMAN: 1,8-bis(dimethyl-amino)naphthalene; HABA: 2-(4-hydroxyphenylazo)benzoic acid; NAD: nicotinamide adenine dinucleotide; ODNs: oligodeoxynucleotides; SA: Sinapinic acid; THAP; 2,4,6-trihydroxyacetophenone.

**Table 2:** Extraction and separation using ionic iquids prior to analysis for matrix assisted laser desorption/ionization mass spectrometry.

ILs	Extraction/Separation Technique	Analytes	Instrumental Parameters	LOD	Conditions	Ref.
CHCAB	DLLME	Phospholipids from soybean	Bruker Daltonics, Nitrogen laser (337 nm) Positive ion mode Acceleration voltage 20 kV Pulse voltage 1300 V Extraction delay time 225 ns 400 laser shots	5 and 18 fmol (LOQ)	5 min extraction time in the presence of 30 mg/mL CHCAB and 1.2% NaCl, using chloroform as an extracting solvent and methanol as a dispersing solvent	[153]
1-alkyl-3-methylimidazolium PF6 (C_n_mim, *n* = 4 and 8) CHCA	LLME	Uranyl nitrate	Bruker Protein-TOF™ Nitrogen laser (337 nm) Pulse width of 3 ns 400 laser shots Both positive and negative modes	0.014–0.098 M	0.1–0.5 M using NaNO_3_ in 1.0 M HNO_3_, TBP (tributyl phosphate) concentration of 1.0 M in the RTILS or in dodecane	[154]
3-methylimidazolium bis[(trifluoromethyl)sulfonyl]amide and 1-butyl-3-methylimidazolium bis[(trifluoromethyl)sulfonyl]amide, 1-hexyl-3-methylimidazolium bis[(trifluoromethyl)sulfonyl] amide and 1-octyl-3-methylimidazolium bis[(trifluoromethyl)sulfonyl]amide		Sr^2+^ and Cs^+^	Voyager DE MALDI–TOF	1.5 mM	1 mL of IL, extracted with 10 mL of cation-containing aqueous solution (1.5 mM) for 60 min in a vibrating mixer.	[155]
PR_4_^+^ cations and ferulate (FA), CHCA, and DHB anions	single-step extraction	Dyes from textiles, malachite green, nile blue nile red, bromothymol blue, fluorescein, kiton red	4800 Plus MALDI–TOF/TOF 200 Hz Nd:YAG laser (355 nm) 400 shots per	0–98%	Samples were centrifuged at 2000 rpm for 30 min, pH 7.5–10, 50–90 °C	[156]
Tetrabutylphosponium chloride IL [Bu_4_P][Cl]	Single-Pot Extraction	dyes associated with structurally robust wool fibers	4800 PlusMALDI–TOF/TOF 200 Hz ND:YAG(355 nm)	0.005 mg of dye per mg of dyed wool into the IL	A cloudy red solution was produced after 24 h. The solution was filtered through a 0.45 µM syringe filter and spotted on the MALDI–MS plate in 1 µL aliquots, either neat or diluted 10,000-fold in methanol	[157]
Platinum nanoparticles mixed 1-butyl-3-methylimidazolium hexafluorophosphate	SDME	*Escherichia coli* and *Serratia marcescens*	Microflex MALDI-MS Nitrogen laser (337 nm) Accelerating voltage of 20 kV 150 laser shots	10^6^cfu mL^−1^	A glass vial was filled with 1 mL of sample solution, spiked with the bacteria; the sample solution was agitated on a magnetic stirrer at room temperature,a 2.0 mL portion of platinum nanoparticles prepared in IL was drawn into a 10 mL microsyringe	[158]
3-Aminoquinoline/CHCA (3AQ/CHCA)	On-target separation	peptides and oligosaccharides	AXIMA-QIT™ Nitrogen laser 337 nm wavelength	5 pmol	Vaporization of water derived from analyte solvent	[159]
Cationic ionic liquid-modified Fe_3_O_4_@SiO_2_ magnetic nanoparticles (CILMS)	Magnetic field	*E. coli*, *Pseudomonas aeruginosa*, and *Staphylococcus aureus*,	Bruker Microflex Nitrogen laser 337 nm wavelength	3.4 × 10^3^, 3.2 × 10^3^, and 4.2 × 10^3^ cfu mL^−1^	<5 min, RT, and use of external magnetic field	[160]
Triethylamine/CHCA	TLC	three arborescidine alkaloids, the anesthesics levobupivacaine and mepivacaine, and the antibiotic tetracycline	Micromass MALDI-TOF Pulse voltage, 2450 V Delay extraction, 100 ns Accelerating voltage, 15 kV Reflectron voltage, 2 kV	5–10 ng	Elution with CHCl_3_/MeOH 9:1	[161]
1-butyl-3methylimidazolium hexafluorophosphate	on-target separation	*Bifidobacterium* *lactis* (Bb12), *Lactobacillus acidophilus* (La5), *Streptococcus thermophilus* and *Lactobacillus bulgaricus* from AB yogurt	Microflex, Bruker Nitrogen laser (337 nm) Accelerating voltages +20 kV Laser energy of 63.2 μJ 200 laser shots	10^7^–10^9^cfu/mL	10 μL of yogurt was added to 100 μL of IL (containing 0.35 mg of AgNPs) and incubated for 10 min before spotting on the MALDI plate.	[129]

**Notes**: DLLME: dispersive liquid-liquid microextraction; LLME: liquid-liquid microextraction; SPE: Single-pot extraction-analysis; TLC: Thin-layer chromatography; LOQ: limits of quantification.
